# Nanopore-m^6^A-finder, a novel m^6^A site caller for Nanopore DRS data

**DOI:** 10.3389/fgene.2026.1770769

**Published:** 2026-04-22

**Authors:** Yuening Yang, Liqun Yu, Li Mo, Changhai Qi, Wei Song, Hua Jin

**Affiliations:** 1 Laboratory of Genetics and Disorders, Key Laboratory of Molecular Medicine and Biotherapy, Aerospace Center Hospital, School of Life Science, Beijing Institute of Technology, Beijing, China; 2 Department of Pathology, Aerospace Center Hospital, Beijing, China; 3 Institute for Precision Medicine, Tsinghua University, Beijing, China; 4 Advanced Technology Research Institute, Beijing Institute of Technology, Jinan, China

**Keywords:** epitranscriptomics, m^6^A, machine learning, Nanopore direct RNA sequencing, poly(A)

## Abstract

**Introduction:**

*N* ^6^-methyladenosine (m^6^A) is a pivotal RNA modification involved in diverse biological and pathological processes. Compared to the m^6^A detection methods based on second-generation sequencing, Nanopore direct RNA sequencing (DRS) offers the unique advantage of capturing native modifications.

**Methods:**

Here, we present Nanopore-m^6^A-Finder (NP-mFinder), a reference-free m^6^A prediction computational framework that employs the XGBoost model in the mRNA exonic region and a hard-voting ensemble of XGBoost and random forest models in the poly(A) region.

**Results and discussion:**

NP-mFinder can determine m^6^A sites as well as estimate their methylation levels from Guppy basecalled DRS data. After training with DRS data of in *vitro*-transcribed RNA, NP-mFinder achieved high performance on held-out test datasets (area under the curve (AUC) ≈0.90; accuracy, precision, recall, and F1-score >0.80). Comparing with canonical m6A detection methods, it recovered 20% of meRIP-seq-defined m6A sites in yeast, and 27% of our HEK293 site prediction overlapped with miCLIP calls. Although single-base overlap with existing DRS-based tools of EpiNano and mAFiA was limited, 73% of our identified m^6^A-containing genes were validated by at least one of them. Benchmarking our method with GLORI v2.0 revealed concordance of 28% at a site level and 85% at a gene level, as well as a mild correlation on m^6^A level estimations. Notably, NP-mFinder achieved 93% precision in detecting m^6^A within the “AAAAA” sequence context in the mRNA exonic region of HEK293T DRS data when compared to high-confidence m^6^A site annotation in GLORI v2.0, demonstrating the good performance of our method in the region possessing a stretch of continuous A-sequences. Moreover, our method predicted that m6A might exist in the human HEK293 poly(A) region, suggesting a possibly conserved phenomenon of a modified poly(A) tail beyond the previously reported T. brucei variant surface glycoprotein (VSG) transcripts. Together, these results established NP-mFinder as a robust and versatile tool for transcriptome-wide m6A profiling with DRS data at single-read resolution.

## 1 Introduction

m^6^A is a biologically important epitranscriptomic modification, installed to mRNAs and noncoding RNAs (ncRNAs) in eukaryotes (Shi et al., 2019). It is predominantly deposited on RNA in the degenerate consensus motif DRACH (where D = A/G/U, R = A/G, and H = A/C/U) by the m^6^A writer complex METTL3–METTL14 (Dominissini et al., 2012; Meyer et al., 2012). However, m^6^A was also found in sites other than the DRACH motif. The hairpin structures in U6 snRNA and the 3′-UTR of MAT2A mRNA were recognized and installed with m^6^A by another m^6^A writer, METTL16 (Chen et al., 2025), demonstrating two different pathways of m^6^A generation (Pendleton et al., 2017; Shima et al., 2017; Ju and Tomita, 2025). These m^6^A sites were specifically identified by the representative reader proteins with YTH domains, including nuclear protein YTHDC1 (DC1) and cytoplasmic proteins YTHDF1/2/3 (DF1/2/3). The binding of m^6^A readers widely regulates the biological processes of target mRNAs, including splicing, translation, and degradation, thus taking part in neural development, cell fate transition, immune response, and DNA repair (Patil et al., 2016; Xiang et al., 2017; Wang et al., 2018; Winkler et al., 2019). Varying disorders appeared once the m^6^A distribution, stoichiometry, or readers changed in cells (Yang et al., 2024).

In the last decade, various methods relying on second-generation sequencing (SGS) have been invented to profile m^6^A sites transcriptome-wide (Yang et al., 2024). The first developed methods, meRIP/m^6^A-seq (Dominissini et al., 2012), miCLIP (Linder et al., 2015), meCLIP (Roberts et al., 2021), and m^6^ACE-seq (Koh et al., 2019), employed immunoprecipitation (IP) with an anti-m^6^A antibody to identify mRNAs carrying m^6^A. Some of these methods could measure the m^6^A site at base resolution. However, these methods are deeply dependent on the quality of the antibody and scarcely quantify the m^6^A level at each site. Later, several enzyme-based m^6^A detection methods were established (Garcia-Campos et al., 2019; Pandey and Pillai, 2019; Zhang et al., 2019). MAZTER-seq/m^6^A-REF-seq can quantify m^6^A level utilizing methylation status-sensitive MazF endoribonuclease only at the sites with an ACA sequence context. eTAM-seq provides both base resolution and stoichiometric information without sequence-context bias (Xiao et al., 2023). However, the requirement of spike-in RNAs and the FTO-treated control group might complicate its experiments. Another method, GLORI, depends on chemical transformation of the installed m^6^A and has improved detection and quantification of the m^6^A sites (Sun et al., 2025).

Different from indirect m^6^A detection with SGS, Oxford Nanopore Technology (ONT) could adopt direct RNA sequencing (DRS) to map m^6^A (Garalde et al., 2018). In theory, DRS could reserve RNA modification information in the sequencing output, raw current intensity signals, which were known as “squiggle”. When an mRNA molecule translocates through a nanopore in DRS, the pore spans approximately five consecutive nucleotides at any given moment. Consequently, each measured ionic current signal at the moment, referred to as an “event”, is jointly influenced by a 5-mer sequence (Jain et al., 2016; McIntyre et al., 2019). In other words, an event represents a successive 5-mer sequence, shifted one nucleotide at a time. For example, the sequence ACGAACU produces three events, corresponding to ACGAA, CGAAC, and GAACU (Jain et al., 2016; McIntyre et al., 2019). Therefore, DRS data combined with computational analysis have the potential to identify modified sites at a single nucleotide (1-nt) resolution by comparing data in two groups, with or without modifications. Several computational methods were developed to profile various RNA modifications. These algorithms utilized differentiated DRS signal patterns between the modified and unmodified ribonucleotides, and the features of basecall error or raw signal differences were used to determine modification status (Yang et al., 2024). EpiNano is based on a basecall error; it assumes that the m^6^A sites are prone to have a higher error rate than normal adenosine (Liu et al., 2019). On the other hand, Nanocompore (Leger et al., 2021), Xpore (Pratanwanich et al., 2021), Tombo, and mAFiA (Chan et al., 2024) are all raw signal-based methods. They find m^6^A sites by discriminating between the differences in raw electronic signals in the DRS recording. These methods are relatively more complex and consume more computing resources. Among them, Xpore and mAFiA can provide the modification levels of m^6^A sites. These methods could examine m^6^A distribution in exonic regions by aligning the DRS data to reference genomes. The analysis of m^6^A in poly(A) tails was not covered in these algorithms.

Recently, it was reported that in addition to mRNA bodies, poly(A) tails also possess m^6^A modifications in *T. brucei,* a protozoan unicellular parasite causing lethal diseases. Surprisingly, half the m^6^A modifications lie in the poly(A) regions of variant surface glycoprotein (VSG) transcripts of *T. brucei* (Viegas et al., 2022). *VSG* genes were activated and highly expressed in blood stream form (BSF) *T. brucei* (Nilsson et al., 2010), and the m^6^A modifications were specifically added to their poly(A) tails co-transcriptionally. A 16-mer motif in their 3′-UTR was responsible for this specificity. In mice, the study using IP with an anti-m^6^A antibody showed that m^6^A did not exist in poly(A) tails (Meyer et al., 2012); however, it is unclear whether m^6^A occurs in poly(A) in some species under specific conditions. Indeed, little attention has been paid to the existence of m^6^A in poly(A) tails. The m^6^A profiles, depending on SGS, could scarcely detect m^6^A in poly(A) because the sequencing reads from the poly(A) region were almost discarded due to not mapping to reference genomes. Considering the current view that the poly(A) tails are not pure and include other ribonucleotides (Chang et al., 2014; Lim et al., 2014; Lim et al., 2016; Lim et al., 2018; Kim et al., 2020), it is worth examining the existence of m^6^A in poly(A).

Here, we developed a new method, Nanopore-m^6^A-Finder (NP-mFinder), which computes Nanopore DRS data and achieves a single nucleotide (1-nt) resolution, site-specific m^6^A level prediction. It applies to exonic regions, including the coding sequences (CDS) and the untranslated regions (UTR) of the transcriptome, without being restricted to the DRACH motif regions. In addition, our method enables profiling m^6^A in the poly(A) region in a reference-free mode and thus gives some insights into further research.

## 2 Results

### 2.1 Find proper binary classification algorithms for m^6^A site detection using Nanopore DRS data

**FIGURE 1:**
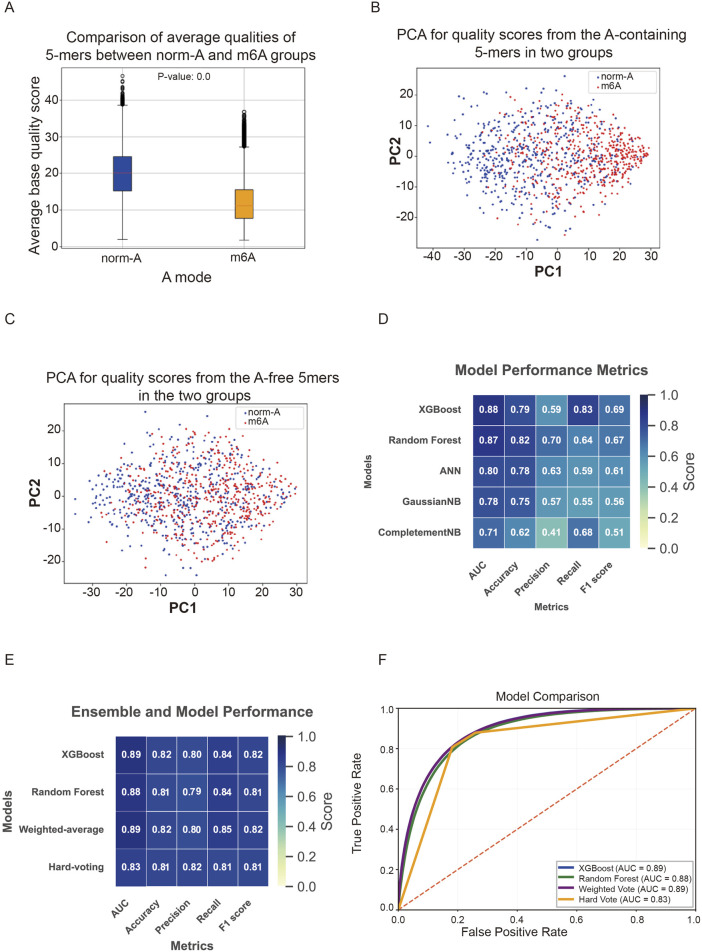
**(A)** Average basecall quality score of 5-mers containing normal adenosine (norm-A) is significantly higher than that of 5-mers containing m^6^A. **(B)** Principal component analysis (PCA) result conducted with the basecall quality scores from adenosine-containing 5-mers (norm-A group, blue dots) and m^6^A-containing 5-mers (m^6^A group, red dots). **(C)** PCA result conducted with the basecall quality scores of the 5-mers, including only the “C”, “G”, and “U” bases from groups of norm-A (blue dots) and m^6^A (red dots). Dot plots in **(B)** but not in **(C)** present a separation trend between the two groups. PC1 = 0.63 and PC2 = 0.19 in **(B)**, while PC1 = 0.56 and PC2 = 0.22 in **(C)**. **(D)** The performance scores of models trained with adenosine-containing 5-mers from the norm-A and m^6^A groups using five different machine learning classification algorithms. The XGBoost and RF models perform better than the other models. **(E)** The performance of the adjusted XGBoost model, the adjusted RF model, and their ensemble models. The adjusted XGBoost model had the best performance, while the accuracy increased a little in the hard-voting ensemble strategy. **(F)** The receiver operating characteristic curves (ROC) from two individually trained models (blue and green lines), and their weighted average ensemble model (purple line) and hard-voting ensemble model (yellow lines). The values of the area under the curve (AUC) are shown. The red dashed line indicates the line y = x (0 < x < 1).

To establish methods for mapping m^6^A at nucleotide resolution, we compared DRS raw data from two groups: the transcripts with or without m^6^A modifications. The computational analysis can be formulated with binary classification algorithms XGBoost, random forest, ANN, SVM, GaussianNB, CompletementNB, etc., thereby classifying a given adenosine into either m^6^A or non-m^6^A. We utilized the raw data of DRS from *in vitro* transcribed (IVT) mRNAs synthesized with normal ATP (norm-A group) or with *N* ^6^-methyladenosine-5′-triphosphate (m^6^A group) (GSM3528752). These synthetic mRNAs were ingeniously designed to comprise all possible 5-mer sequence combinations, while minimizing RNA secondary structure, with ∼10 times median occurrence frequency of each 5-mer in IVT mRNAs (Liu et al., 2019). Based on the knowledge that the modification of a certain nucleotide will influence the current intensity of the 5-mer containing it, we compared signal perturbations between the groups of “norm-A” and “m^6^A”. We first extracted all events from each read using a sliding window of length 5 nucleotides (5-mer) with a step size of 1. Then, the 5-mers containing at least one adenosine were picked out individually in two groups, including 667,245 events in the m^6^A group and 1,629,669 events in the norm-A group. We further extracted information about the corresponding bases, quality scores, and signal traces for all these 5-mers presented in the TSV form of the alignment SAM file. As expected, the average quality scores of 5-mers in the m^6^A group were significantly lower than those in the norm-A group (Figure 1A). Principal component analysis (PCA) of quality scores was conducted to examine the separation of the selected 5-mers containing adenosine into two groups. The analysis showed that the quality scores of 5-mers tend to separate into two groups (Figure 1B); in contrast, the quality scores of the 5-mers only including “C”, “G”, and “U” bases did not exhibit a trend of separation between the norm-A and m^6^A groups (Figure 1C). Aside from basecall quality, we found that the basecall feature of Trace indicates the likelihood of a given nucleotide being identified as one of the four types: “A”, “C”, “G”, or “U”. Therefore, we utilized the nucleotide identities, along with quality scores and trace values of the 5-mers, as features to train machine learning classification models.

Subsequently, several binary classification algorithms were applied to predict m^6^A. We trained and tested the performance of six different machine learning algorithms, XGBoost, Random Forest, ANN, SVM, GaussianNB, and CompletementNB, in classifying two categories: the adenosine-containing 5-mers from the norm-A group and those from the m^6^A group. These algorithms possess different characteristics (Table 1). Because the SVM algorithm was too slow when processing large datasets and failed to produce the final results, the performance of five algorithms with the training dataset was obtained (Figure 1D). Key metrics, including accuracy, precision, recall, and F1-score of models, indicated the model performance. Among these models, we chose the XGBoost and random forest (RF) models, as they performed best with the training dataset. Then, more training data were added by randomly sampling the remaining DRS data of IVT transcripts (SRR8767350 and SRR8767351), and data entries were balanced to the numbers of 991,512 in a norm-A (n = 991,512) and m^6^A (n = 991,512) training dataset. Further adjustment of algorithm parameters led to better performance of the XGBoost and RF models (Figure 1E). To enhance prediction performance, we also tried XGBoost-RF ensemble strategies, the weighted average method, and the hard-voting method. The weighted average ensemble method, which was performed with a parameter of weighted XGBoost 0.5 and weighted RF 0.5, demonstrated similar performance metrics as the XGBoost model only. The XGBoost-RF hard-voting ensemble method exhibited slightly better accuracy in identifying 5-mers containing m^6^A, even though this improvement sacrificed other performance metrics (Figure 1E). The receiver operating characteristic (ROC) curves of these four methods also revealed that their true positive rates were dramatically higher than their false positive rates (Figure 1F).

**TABLE 1:** Characteristics of six machine learning classification algorithms.

Algorithm	Noise robustness	Sparse feature	Independent feature	Discrete feature	Data size	Parameter tuning
XGBoost	Average	Good	Not required	Not required	Large and fast	Complex
Random forest	Good	Average	Not required	Not required	Large but time consuming	Simple
ANN	Parameter dependent	Average	Not required	Not required	Large but time consuming	Simple
SVM	Good	Good	Not required	Not required	Small	Complex
GaussianNB	Average	Average	Required	Continuous feature required	Small	Simple
CompletementNB	Average	Average	Required	Required	Small	Simple

**FIGURE 2:**
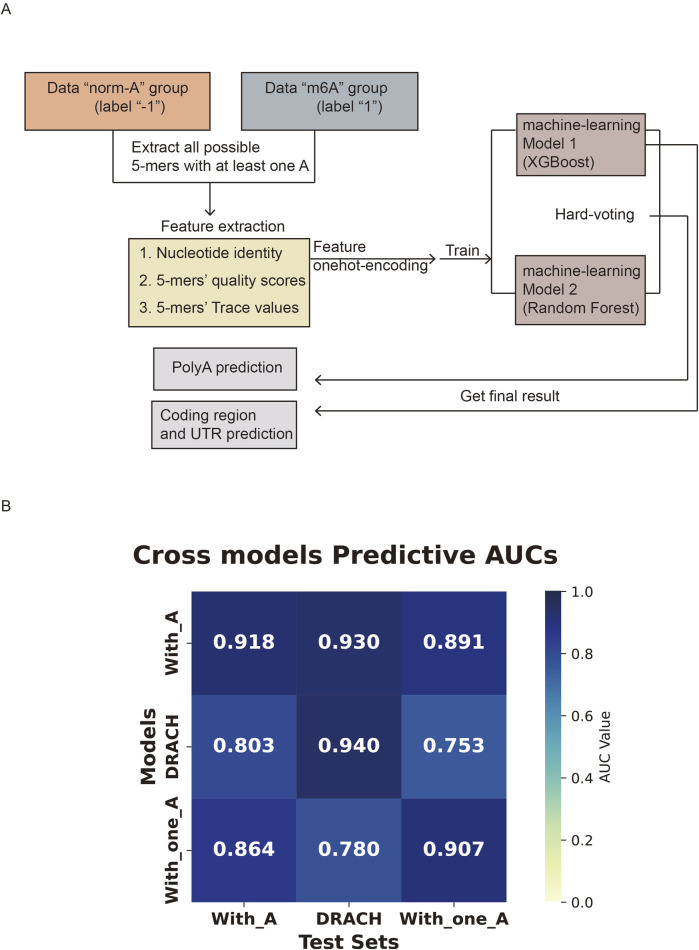
Heatmaps for model performance. **(A)** Pipeline of training two models and the prediction of 5-mers with m^6^A. **(B)** The heatmap shows the cross-validation of XGBoost models trained on different 5-mer subsets of IVT mRNA DRS. The models were trained using three different classes of 5-mers: the “with_A” class containing one or more adenosines, the “DRACH” class including the DRACH motif, and the “with_one_A” class containing only one adenosine within the 5-mers. The x-axis indicates the test datasets, and the y-axis shows the corresponding training sets. Each cell displays the AUC score (color and number) for the model tested on a given dataset.

According to the above test, we established the workflow for model training and prediction as illustrated in Figure 2A. The adjusted XGBoost model was used to predict m^6^A later from the mRNA exonic regions of endogenous transcripts, including coding sequence and untranslated region (CDS and UTR). Meanwhile, the XGBoost-RF hard-voting ensemble model was employed to assess m^6^A status in poly(A) tails of endogenous transcripts. Considering that the model will be applied to the *in vivo* is likely to contain only a single modified adenosine, we next evaluated our model performance in 5-mers containing only one adenosine. To do this, we selected 5-mers further with two specific categories from the DRS of IVT transcripts: 5-mers containing only one adenosine, and 5-mers matching the DRACH motif. When models trained and tested with 5-mers in the same category, they performed well (Figure 2B). Cross-validation between different categories revealed that the model trained on 5-mers containing one or more adenosines performed equally well across specialized subsets (Figure 2B). This indicated that the model trained on 5-mers with one or more adenosines demonstrated the best generalizability for predicting m^6^A.

### 2.2 Using the trained XGBoost model can predict m^6^A sites and levels in the mRNA exonic regions with good performance

Next, we aimed to assess the accuracy of our models in predicting two *in vivo* datasets. We downloaded DRS data from wild type (WT) and *ime4* knockout (*ime4*Δ) budding yeast SK1 (Liu et al., 2019), as well as the METTL3 knockdown (shMETTL3) and its WT human HEK293 cell lines (Lorenz et al., 2020). The homologous genes *ime4* and *mettl3* were known to be m^6^A writers in yeasts and mammals, respectively. Consequently, the *ime4*Δ yeast strain and the shMETTL3 HEK293 cell line are expected to exhibit no m^6^A modifications in their transcriptomes.

**FIGURE 3:**
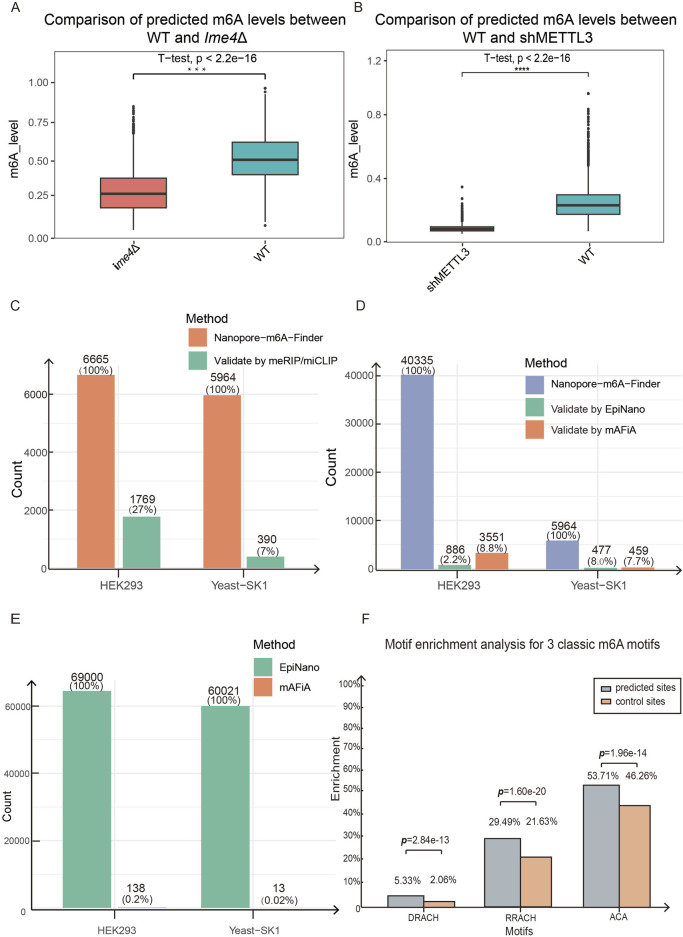
**(A)** Methylation levels were compared between yeast WT and *ime4*Δ strains in the 5,964 m^6^A sites identified by NP-mFinder. The m^6^A levels in WT are significantly higher than in *ime4*Δ. *N = 3*. **(B)** Methylation levels were compared between human HEK293 WT and shMETTL3 cells in the 40,335 m^6^A sites identified by NP-mFinder. The m^6^A levels of WT are significantly higher than those of the shMETTL3 cells. *N* = 1. **(C)** Counts and percentages of the NP-mFinder-identified m^6^A sites were validated by traditional IP-based meRIP or miCLIP methods. **(D)** Counts and percentages of the NP-mFinder-identified m^6^A sites were also detected by other DRS m^6^A callers. EpiNano and mAFiA validated some NP-mFinder sites, while our method found many new unique sites. **(E)** Comparison of EpiNano and mAFiA in detecting the potential m^6^A sites in HEK293 and yeast SK1. Only 0.2% of the EpiNano sites in HEK293 were also detected by mAFiA, and 0.02% of the EpiNano sites in yeast SK1 were examined by mAFiA. **(F)** Motif enrichment analysis found that three classic m^6^A motifs were significantly enriched in our 5,964 yeast m^6^A sites compared with random control sites.

In the yeast data, our XGBoost model identified 5,964 m^6^A sites based on three replicates of the WT and *ime4*Δ DRS data (Supplementary Table S1). The m^6^A level at a given adenosine site was defined as the portion of RNA molecules (reads) that were predicted to have a modification at the site. The average m^6^A levels of the identified 5,964 sites were significantly higher in the WT yeast strain than in the *ime4*Δ strain (Figure 3A). Among these XGBoost-identified sites, 390 sites overlapped with 1,308 meRIP m^6^A sites (Schwartz et al., 2013) (Figure 3C), accounting for 20.03% of the 1,308 meRIP sites. We compared our 5,964 sites with the prediction results from two other DRS m^6^A detection methods, EpiNano_Differ (Liu et al., 2019) and mAFiA. Our NP-mFinder-predicted sites had cross-validation rates of 8% with EpiNano_Differ and 7.7% with mAFiA (Figure 3D). We also compared the yeast m^6^A sites detected using EpiNano_Differ with those validated by mAFiA. Among the 60,021 sites identified by EpiNano_Differ, only 13 (0.02%) were supported by mAFiA, which was much lower than the cross-validation rates of our NP-mFinder (Figure 3E).

In HEK293, our method identified 40,335 sites based on the WT and shMETTL3 DRS data (Supplementary Table S2). The average m^6^A levels of these sites were significantly higher in WT cells than in shMETTL3 cells (Figure 3B). Among our predicted sites, 6,665 sites were covered by input RNA-seq data from the miCLIP experiment (Linder et al., 2015), and 1,769 sites overlapped with the m^6^A sites detected by miCLIP (Figure 3C), which validated 26.5% of the covered sites. When comparing our method with EpiNano_Differ and mAFiA prediction in HEK293, we found 886 (2%) and 3,551 (8.8%) sites were validated by these two methods, respectively (Figure 3D). In contrast, comparing EpiNano to mAFiA, only 138 (0.2%) of EpiNano-detected sites were validated by the mAFiA method, which means the site cross-validation is generally low between these two methods (Figure 3E). Cross-validation of our method with these two methods (Figure 3D) performed better than the validation between EpiNano and mAFiA (Figure 3E). Additionally, we utilized MEME’s simple enrichment analysis to evaluate whether the classical m^6^A motifs were more enriched within the ±4-bp regions surrounding the predicted 66,026 sites compared to the same number of non-predicted control sites. The results revealed significant enrichment of all three classic m^6^A motifs, DRACH, RRACH, and ACA, within these regions (Figure 3F).

**FIGURE 4:**
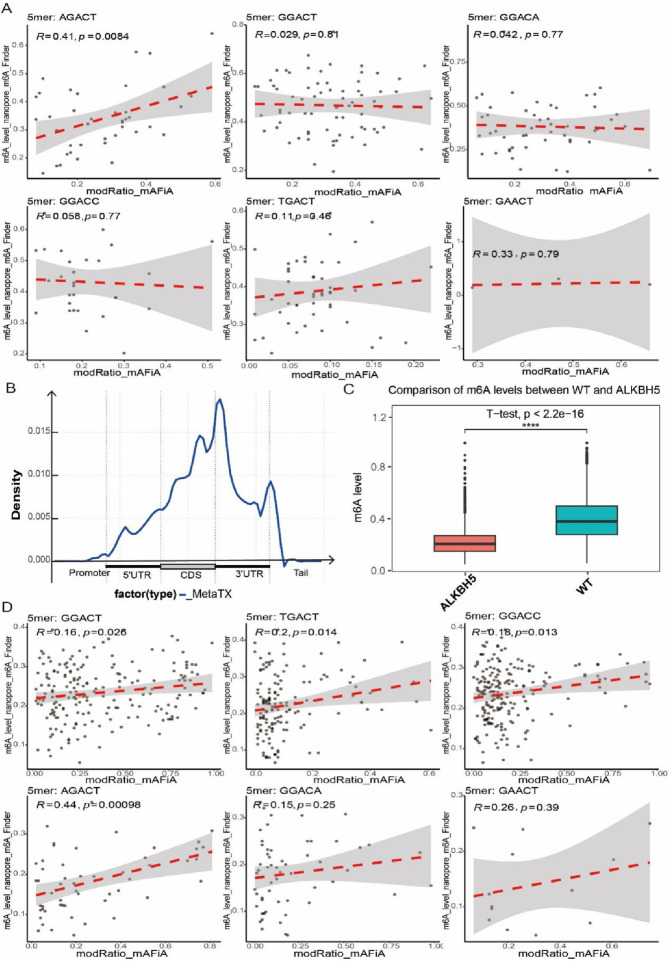
**(A)** Pearson correlation of predicted m^6^A levels between shared m^6^A sites (sample size: 240) detected by both NP-mFinder and mAFiA in yeast. **(B)** Metagene graph of NP-mFinder-detected m^6^A sites on human gene structure. The peak was in the 3′-UTR near the stop codon. **(C)** Validation of our identified 40,335 m^6^A sites in HMEC WT and eraser ALKBH5 overexpression cells. The m^6^A levels of these sites are significantly higher in WT than in ALKBH5 overexpression cells. **(D)** Pearson correlation of identified m^6^A levels between the shared m^6^A sites (sample size: 670) detected by both NP-mFinder and mAFiA in human HEK293.

**FIGURE 5:**
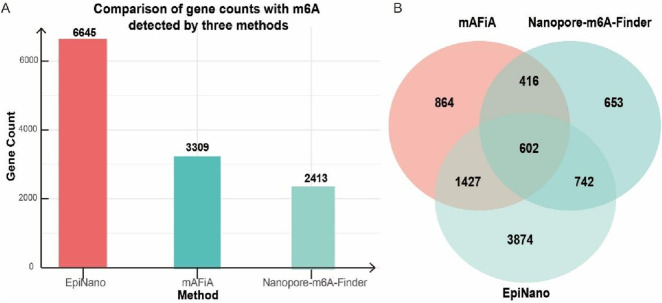
**(A)** Numbers of m^6^A-containing genes identified by three independent methods: EpiNano, mAFiA, and NP-mFinder. **(B)** The Venn graph shows the common m^6^A-containing gene counts identified by three independent methods: NP-mFinder, mAFiA, and EpiNano.

Because mAFiA can predict the m^6^A level of each site, we conducted quantification correlation analysis for the overlapped 240 m^6^A sites between mAFiA and our method in yeast. The result showed that only under the m^6^A motif of AGACT, two methods had a mild positive correlation in m^6^A level prediction (Figure 4A). We then carried out metagene analysis and plotted our discovered 40,335 m^6^A sites to their relative positions on gene structure, and m^6^A peak positions appeared on the 3′-UTR around the stop codon (Figure 4B). This is highly consistent with the previous findings (Dominissini et al., 2012; Meyer et al., 2012). We also checked these sites in DRS data from the m^6^A-eraser ALKBH5-overexpressed HMEC cell line (Lorenz et al., 2020), and we found that these sites had significantly decreased m^6^A levels in the eraser overexpression cells compared with WT cells (Figure 4C). This also supports the accuracy of our method. Similarly, quantification analysis was conducted for the 670 overlapped m^6^A sites between our method and mAFiA in HEK293, which revealed that the quantification of the two methods had a weak positive correlation under the m^6^A motifs of GGACT, TGACT, GGACC, and AGACT (Figure 4D). We next investigated the overlapped genes with m^6^A modification in HEK293. EpiNano, mAFiA, and NP-mFinder identified 6645, 3309, and 2413 genes with m^6^A, respectively (Figure 5A). Surprisingly, we found the m^6^A genes detected by our NP-mFinder were validated by two other methods to approximately 73%, which further confirmed the accuracy of our method (Figure 5B).

Together, these findings underscored that NP-mFinder achieved markedly great consistency with independent approaches, meanwhile revealed new candidate sites, supporting its utility as an accurate rather than merely a novel method for m^6^A detection.

**FIGURE 6:**
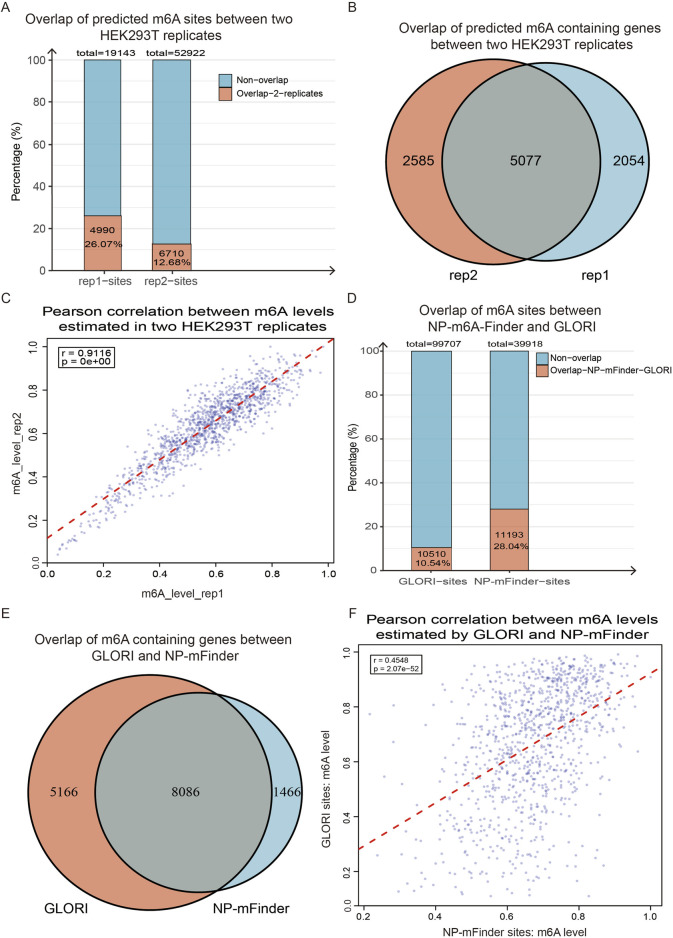
**(A)** Overlap of m^6^A sites identified by NP-mFinder in two independent Nanopore DRS replicates from HEK293T cells (Chan et al., 2024). Replicate 1 (rep1) yielded 19,143 m^6^A sites, and 26% of them were also detected in replicate 2 (rep2); rep2 identified 52,922 sites, with 12.7% of them also determined in rep1. **(B)** Concordance of m^6^A-containing genes between two DRS replicates. A total of 7,131 and 7,662 m^6^A-containing genes were called in rep1 and rep2 by NP-mFinder, and substantial overlap between them indicated high reproducibility at the gene level. **(C)** Pearson correlation of m^6^A modification levels between two replicates. A strong positive correlation (*r* = 0.91, *p* < 10^−15^, sample size: 1,234) demonstrated the quantitative consistency of NP-mFinder across biological replicates. **(D)** Cross-method comparison of m^6^A sites identified by NP-mFinder and GLORI v2.0 (Sun et al., 2025). GLORI v2.0 reported 99,707 high-confidence m^6^A sites, while NP-mFinder predicted 39,918 sites; 28% of NP-mFinder sites overlapped with GLORI annotations. **(E)** Overlap of m^6^A-modified genes between NP-mFinder and GLORI v2.0. In the genes called by NP-mFinder, 85% was also identified by GLORI v2.0, highlighting strong agreement at the gene level. **(F)** Pearson correlation of m^6^A levels at the sites jointly identified by both methods. Statistically significant positive correlation was observed (*r* = 0.455, *p* = 2.07 × 10^−52^, sample size: 1,004), supporting the biological coherence of NP-mFinder prediction with orthogonal experimental data.

To further evaluate the robustness and accuracy of NP-mFinder, we downloaded two biological replicates of the Nanopore DRS data of HEK293T WT and METTL3 knockout (KO) cells from the European Bioinformatics Institute (EBI) (Chan et al., 2024). These datasets were generated using the SQK-RNA002 library preparation kit and R9.4.1 flow cells, and the conditions were fully compatible with our training data and the Guppy basecaller version used in our model development. We applied NP-mFinder to both replicates and assessed the concordance of predicted m^6^A sites. Approximately 26% of the sites called in replicate 1 (rep1) were also detected in replicate 2 (rep2), while ∼13% of rep2 sites overlapped with those in rep1 (Figure 6A). At the gene level, the overlap of m^6^A-modified genes between two replicates reached ∼70% (Figure 6B). Moreover, the methylation levels of the sites consistently identified by both replicates exhibited a strong positive correlation (Pearson’s *r* = 0.91), underscoring the reproducibility of our method (Figure 6C).

**FIGURE 7:**
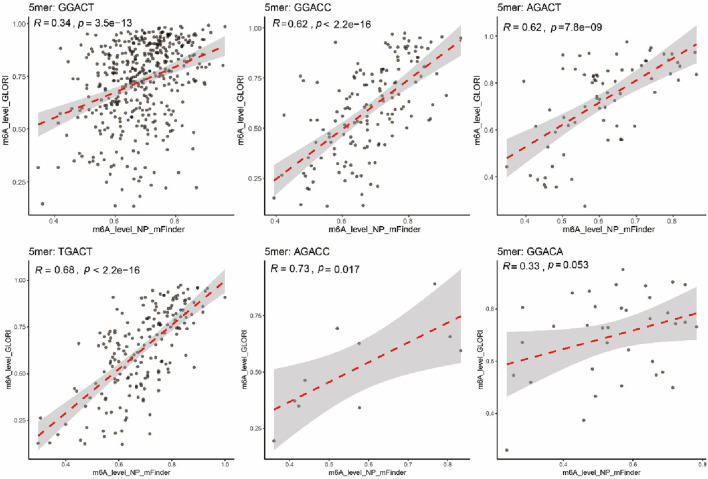
Pearson correlation of the m^6^A levels between the common m^6^A sites (sample size: 894) detected by NP-mFinder and GLORI v2.0 in human HEK293T under different DRACH sequence contexts.

To benchmark against an orthogonal experimental approach, we compared our prediction with the high-confidence m^6^A map of HEK293T from GLORI v2.0, a chemically refined, degradation-minimized protocol that offers improved speed and accuracy compared to its earlier version (Sun et al., 2025). Among the 99,707 m^6^A sites reported by GLORI v2.0, 28% of NP-mFinder sites overlapped with the GLORI sites (Figure 6D). Notably, at the gene level, 85% of the NP-mFinder genes overlapped with GLORI v2.0 (Figure 6E). Furthermore, methylation levels at the sites called by both methods showed moderate but significant positive correlation (Pearson’s *r* = 0.455) (Figure 6F). In addition, under certain DRACH sequence contexts, the correlations of m^6^A levels in NP-mFinder and GLORI v2.0 methods were stronger; the correlation efficiency reached 0.62–0.73 in particular motifs (Figure 7), pointing out biological coherence in different m^6^A profiling strategies. Collectively, these cross-replicate and cross-method validation analyses demonstrated that NP-mFinder is a stable, accurate, and biologically relevant tool for transcriptome-wide m^6^A site detection from Nanopore DRS data.

### 2.3 m^6^A was detected in mRNA poly(A) tails in *T. brucei* and human cells by our XGBoost-random forest (RF) hard-voting ensemble model

**FIGURE 8:**
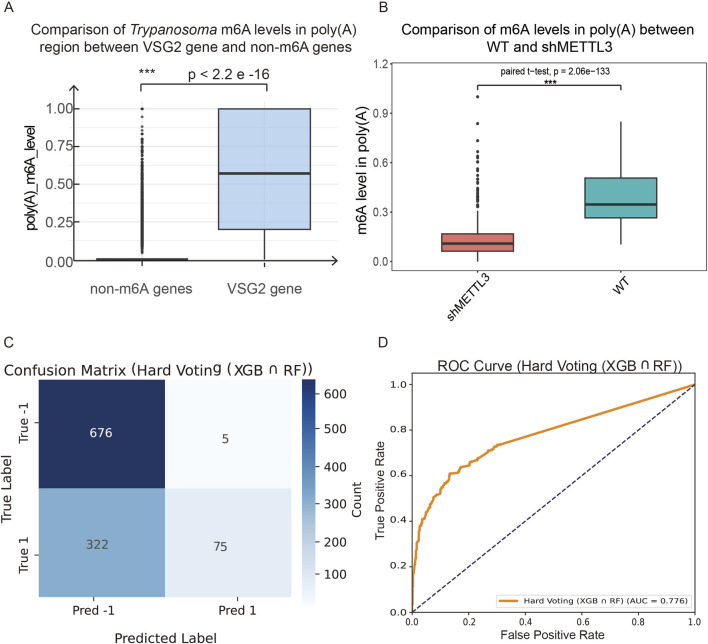
**(A)** According to predication of our XGBoost-RF hard-voting ensemble model, VSG2 transcripts, the well-known gene with m^6^A-modified poly(A) tails in *Trypanosoma* (Viegas et al., 2022), have significantly higher poly(A) m^6^A levels than the transcripts known to be without m^6^A in poly(A) (2,693 positive samples and 26,609 negative samples using Student *t*-test). **(B)** Comparison of poly(A) m^6^A levels between the genes from HEK293 WT and shMETTL3 (Lorenz et al., 2020). The poly(A) m^6^A levels of WT are significantly higher than those of shMETTL3 (paired Student *t*-test; sample size: 552 each group). **(C,D)** The m^6^A sites in “AAAAA” sequence context were selected from the HEK293T results of GLORI version 2 (Sun et al., 2025). Then, the sites with m^6^A levels higher than 70% were further selected from these sites. We next extracted 5-mers covering these sites from the nanopore DRS HEK293T data (WT sample size 397 and Mettl3-KO sample size 681) (Chan et al., 2024) to validate the prediction performance of our XGBoost-RF hard-voting ensemble model in the “AAAAA” context from the mRNA exonic region. The confusion matrix **(C)** and ROC curve **(D)** are shown.

We then applied our trained models to predict m^6^A modifications within the poly(A) regions of transcripts from human HEK293 cells. Prior to the analysis, we validated the model performance specifically within poly(A) tails to ensure accuracy. To enhance prediction accuracy, we employed a hard-voting ensemble approach combining XGBoost and RF classifiers (Figure 2A). A previous report found m^6^A in the poly(A) tails of *T. brucei* VSG transcripts. So, 2,693 of VSG2 transcripts were utilized as positive controls. For negative controls, we selected 26,609 transcripts previously shown to lack m^6^A in their poly(A) (Viegas et al., 2022). Similar to the quantification of m^6^A level in the mRNA exonic region, we defined poly(A) modification level as the number of predicted m^6^A-containing 5-mers divided by the poly(A) tail length. Application of our XGBoost-RF hard-voting ensemble model revealed that VSG2 poly(A) tails exhibited significantly higher predicted poly(A) m^6^A levels than those of the control transcripts using a *t-*test (Figure 8A).

Next, we applied the models to predict m^6^A in the HEK293 poly(A) region (Lorenz et al., 2020). For HEK293 cells, we removed the genes with coverage less than 20 in DRS and calculated the average m^6^A level of poly(A) for each gene. We identified that 580 genes had m^6^A in the poly(A) of WT. Among them, 552 genes were detected in the shMETTL3 group with intact poly(A) tails (Supplementary Table S3). These 552 genes in WT cells had significantly higher poly(A) m^6^A levels than the shMETTL3 cells using the paired *t*-test comparison. It provided the possibility that m^6^A modification existed in human mRNA poly(A) tails and was predicted to be installed in the poly(A) region by METTL3, the classic m^6^A writer (Figure 8B).

To further support the prediction precision of our ensemble model as well as the hypothesis of the existence of m^6^A in mammalian poly(A) region, we additionally verified the predictive performance of our ensemble XGBoost-RF hard-voting model for identifying m^6^A within successive 5-mer A-sequences from the mRNA body/exonic region. We focused on a high-confidence subset of m^6^A sites from the sequence context of “AAAAA” in the mRNA exonic region. Specifically, from the 99,707 m^6^A sites annotated by GLORI v2.0 in HEK293T (Sun et al., 2025), we first identified 1,374 sites residing in an “AAAAA” sequence context, and further filtered these to retain 243 high-confidence sites with m^6^A levels higher than 70%. Using Nanopore DRS data from HEK293T WT and METTL3-KO cells downloaded in EBI (Chan et al., 2024), we extracted all 5-mers spanning GLORI high-confidence “AAAAA” sites. We gathered 681 5-mers from the METTL3-KO sample (labeled as “True −1”) and 397 5-mers from the WT (labeled as “True 1”). When we applied these 5-mers to our integrated NP-mFinder model, the prediction achieved the accuracy of 0.70, precision of 0.94, recall of 0.19, F1-score of 0.31 (see confusion matrix in Figure 8C), and the AUC of 0.78 (see ROC curve in Figure 8D). The high-precision prediction of “A” 5-mers in the mRNA exonic region confirmed that our integrated NP-mFinder can also reliably identify true m^6^A-containing “AAAAA” from an exonic region and supported our hypothesis regarding m^6^A in the polyA region. Due to the limited sensitivity, a fraction of m^6^A may remain undetected. Therefore, although NP-mFinder precluded precise quantification of m^6^A stoichiometry in the repetitive A-sequence region, these results predicted the presence of m^6^A in the poly(A) tails of HEK293 transcripts.

## 3 Discussion

ONT direct RNA sequencing preserves the native features of RNA molecules, providing the possibility of discriminating m^6^A from norm-A at the nucleotide level. Current methods for detecting RNA modifications predominantly rely on SGS, which rarely reaches a single-molecule level with quantitative information on potential m^6^A sites. Recent research has highlighted that a modification at a given site does not occur in every transcript containing it, and the modification percentage or level is believed to be crucial for molecular fate.

The Nanopore-m^6^A-Finder (NP-mFinder) relies on a machine learning classifier to discriminate m^6^A sites by using Guppy basecaller’s output as features. We trained and tested six binary classification algorithms using IVT mRNA DRS data from norm-A and m^6^A groups. The features of nucleotide identity, quality score, and trace value of 5-mers possessing at least one adenosine were used for training and testing the models. Among them, XGBoost and RF models performed the best (Figure 1D). Further improvement was carried out by balancing data entries between norm-A and m^6^A, as well as by adjusting algorithm parameters. We also established XGBoost-RF ensemble models employing the weighted average method and the hard-voting strategy. All these adjusted models and the ensemble models worked excellently, as shown by performance metrics (Figure 1E).

The adjusted XGBoost model was used for m^6^A mapping in the mRNA exonic regions of endogenous transcripts, using the datasets from m^6^A-writer-impaired yeast and mammalian cells. Our NP-mFinder recovered 20% of the m^6^A sites from yeast meRIP-seq, and 26.5% of our predicted HEK293 sites overlapped with the miCLIP result, demonstrating measurable concordance with antibody-based approaches (Figure 3C). In addition, our m^6^A sites were enriched in the near downstream of the stop codon (Figure 4B). The consistency of mAFiA, EpiNano, and our method at a site level was not good (Figures 3D–F), but 73% of our identified m^6^A-possessing genes were validated by mAFiA or EpiNano (Figure 5B). Further cross-method validation of our NP-mFinder with GLORI v2.0 showed that the m^6^A sites and genes detected by our method overlapped with GLORI v2.0 at percentages of 28% and 85%, supporting the accuracy of our method (Figures 6D,E). Moreover, our m^6^A level prediction had a moderate yet significant positive correlation with GLORI v2.0 (*r* = 0.455), especially in particular DRACH sequences (Figures 6F, 7).

When the XGBoost-RF ensembled model was employed for m^6^A prediction in mRNA poly(A) tails, VSG2 transcripts, the well-known *T. brucei* genes with m^6^A-including poly(A) tails (Viegas et al., 2022), had significantly higher poly(A) m^6^A levels than the other transcripts (Figure 8A). Interestingly, comparing poly(A) m^6^A levels between HEK293 WT and shMETTL3 cells showed that the poly(A) m^6^A levels of WT were significantly higher than those of the m^6^A-writer-depleted cells. This raised the possibility that human cells might have m^6^A in poly(A) tails (Figure 8B). In order to demonstrate that our ensembled model can properly learn m^6^A status in successive A-sequence regions, we further extracted high-confidence m^6^A sites from the mRNA exonic region with an “AAAAA” context in GLORI v2.0 results (Sun et al., 2025). The same 5-mers were extracted from HEK293T DRS data (Chan et al., 2024) and used to evaluate our hard-voting ensembled XGB-RF model, which confirmed our method also works well in “AAAAA” sequences from exonic regions with a precision of 93% (Figures 8C,D).

It should be mentioned that there are some shortcomings to our method. First, although our method can provide single-base resolution of m^6^A detection, the site-calling precision depends heavily on the data size. More sequencing depth may bring more precise site calling. Second, our method could only predict relative m^6^A levels among samples with different biological backgrounds (Figures 3A,B; Figure 4C) and had some agreement with GLORI v2.0 quantification (Figures 6F, 7). However, the m^6^A quantification still varied largely among different methods; it is not yet accurate and requires continuous improvement. Third, our method relies on Guppy basecaller features; the training data were based on the SQK-R001 kit and the R9.4.1 flow cell. This makes our method compatible only with the SQK-RNA002 (R002)-derived data we tested (Figure 6), but not with the latest R004 data. Nevertheless, given the abundant publicly available R002 datasets, our models will be very useful for monitoring m^6^A in different biological contexts based on these data, enabling the recovery of epitranscriptomic insights. It will be very great to develop the second version of NP-mFinder compatible with the latest Dorado basecaller and R004 datasets in the future.

In this study, we presented the NP-mFinder method for m^6^A mapping. In two other m^6^A-caller methods, EpiNano_Differ requires a reference genome for read alignment, and mAFiA additionally requires an input of potential m^6^A candidate sites for validation. Compared to them, our method enables m^6^A detection in each RNA molecule without the need for a reference genome or pre-annotated potential m^6^A sites. NP-mFinder can detect m^6^A sites *de novo*, free from any sequence-context bias. It can also identify m^6^A status in the poly(A) region, which lacks a reference genome. Additionally, the hardware requirements for our method are more economical than those relying on deep learning models.

## 4 Conclusion

To conclude, we developed a single-molecule resolution, reference-independent method to call the m^6^A sites and report their modification levels. The method can provide the m^6^A profile of the transcriptome in both the mRNA exonic region and the poly(A) tail region. Our method will help researchers in understanding the m^6^A status in the transcriptome.

## 5 Methods

### 5.1 Basecalling and mapping

Reads were locally basecalled using Guppy 6.0.1 (ONT) with parameter–fast5_out to save the fast5 files for individual reads. Minimap2 was used for quality filtered reads’ mapping with the settings -ax map-ont. Sequence alignment/map (SAM) alignment files were converted to tab-delimited format using sam2tsv version 1.0 from jvarkit. The reference sequence of the reads were four synthetic sequences (curlcakes), yeast SK1 genome and transcriptome, and human hg38 genome and cDNA respectively.

### 5.2 Feature extraction and encoding

For model training and model prediction, we extract quality and trace information of each 5-mer containing “As” from individual reads presented in the TSV form of the alignment SAM file. The h5py (version 3.13.0) module in Python was used to parse each individual fast5 file. For each 5-mer containing adenines, we extracted the corresponding bases, quality scores, and signal traces. In addition, the genomic positions of the 5-mers were recorded using in-house Python scripts (available on GitHub). The extracted features were then processed by one-hot encoding or segmentation for model training. We use XGBoost (version 1.6.0) and RandomForestClassifier from the sklearn module (version 1.7.0) to train our XGBoost model and random forest model. All in-house Python scripts used to extract the features above are publicly available as part of Nanopore_m^6^A_Finder on GitHub.

### 5.3 Machine learning

We downloaded the Nanopore DRS data of the *in vitro*-transcribed transcripts from the GEO database with accession number GSM3528752 (Liu et al., 2019). The data were labeled with “with m^6^A” and “without m^6^A” for the difference in their *in vitro* transcription process.

The extracted features from both m^6^A-modified and -unmodified “curlcakes” were used as input to train XGBoost and random forest models. Initial training (80% of the 5-mers) and testing (20%) of the XGBoost model or RF model were performed with m^6^A-modified and unmodified “curlcake” reads from SRR8767349 and SRR8767348. Multiple XGBoost parameter sets were tested, and the best-performing set was: objective = binary:logistic; eval_metric = [auc, logloss]; learning_rate = 0.1; max_depth = 8; min_child_weight = 1; subsample = 0.8; colsample_bytree = 0.7; tree_method = hist; scale_pos_weight = ratio of negative to positive samples. For the random forest model, the only parameter specified was the number of decision trees, which was set to 100. The trained models were validated on independent sequencing runs of m^6^A-modified and unmodified “curlcakes” (SRR8767351 and SRR8767350), which were not used during initial training or testing. Moreover, we tested whether hard voting or weighted voting (with a 1:1 ratio between the two models) could improve performance. The codes for building the two machine learning models and ensemble strategy tests are publicly available on GitHub. The accuracy of the models has been computed as the sum of the correct m^6^A modification predictions that were correctly predicted m^6^A-modified k-mers (TP) and correctly predicted unmodified k-mers (true negatives, TN) divided by the total number of k-mers tested. We should note that a limitation in utilizing “curlcakes” to generate all possible 5-mers is that 5-mers that contain more than one “A” will contain more than one modification in the k-mer; for example, AGACC will, in fact, be m^6^AGm^6^ACC; however, such 5-mers are unlikely to occur in a biological context. Therefore, we also trained XGBoost models for 5-mers with only 1 A and with A in the middle position of a context of DRACH motif. We compared the generalization capability of the three XGBoost models trained on different datasets. We decided that the model trained with “with A” data is the best.

### 5.4 Prediction of m^6^A-modified sites and levels in coding regions and UTRs of yeast, human HEK293 cells, and human HEK293T cells using the trained XGBoost model

Sequenced reads from six yeast sample (GSE126213) (Liu et al., 2019), two human HEK293 sample (GSE132971) (Lorenz et al., 2020), and four human HEK293T samples (WT-rep1 and WT-rep3: PRJEB74106, Mettl3-KO-rep1 and Mettl3-KO-rep2: PRJEB40872) (Chan et al., 2024) were aligned to their respective reference genomes. Coding regions and UTRs were subsequently extracted from the aligned reads and used for 5-mer feature derivation. These 5-mers were predicted to be m^6^A-modified or -unmodified; the position of the As in the 5-mers was recorded and referred to genomic positions using in-house Python scripts in a strategy of piling up. We then compared the predicted m^6^A levels in WT and in m^6^A writer knockout (KO) or knockdown (KD) samples. We retained only the sites whose m^6^A levels in the WT were significantly higher than those in the KO or KD samples, as determined by the Wilcoxon test. A total of 5,964 sites were identified from the three yeast WT samples and three ime4Δ sample; 40,335 sites were identified from the human HEK293 WT cells and shMETTL3 cells; and 39,918 sites were identified from the human HEK293T WT cells and METTL3 knockout cells.

### 5.5 Analysis of the identified sites

m^6^A levels were calculated by dividing the number of reads predicted to harbor m^6^A at a given site by the total read coverage at that site. The yeast’s 5,964 identified m^6^A sites and 5,964 control sites were sent to MEME’s SEA assay using default settings on https://meme-suite.org/meme/. We compared the identified yeast m^6^A sites with the 1308 meRIP-identified m^6^A sites (Schwartz et al., 2013) within an upstream and downstream 100 bp regions. We also compared the identified human HEK293 cells’ m^6^A sites with the sites we found by PureCLIP software (https://github.com/skrakau/PureCLIP) from miCLIP data (Linder et al., 2015) within 20-bp upstream and downstream regions. In addition, we used the same dataset from yeast and human HEK293 cells to run EpiNano_Differ (https://github.com/enovoa/EpiNano) and mAFiA (Chan et al., 2024) and then compared the m^6^A sites identified by our models to the results of the above two methods. We also compared the m^6^A-site contained genes from the three prediction methods in HEK293 cells. In addition, we used the R script package MetaTX to plot the metagene graph for the 40,335 identified m^6^A sites in HEK293 cells. We compared the m^6^A levels of the sites identified in HEK293 cells to those of the eraser overexpression HMEC cells. We used Pearson correlation analysis to determine whether the m^6^A levels of the common sites from the two methods are positively correlated. Furthermore, we compared the robustness of NP-mFinder by comparing the detected sites and methylation levels from two HEK293T replicates. All the overlap sites were calculated by expanding the sites to a 20 bp up- and downstream window. We also compared our m^6^A sites to GLORI v2.0 sites (Sun et al., 2025). The overlap sites were similarly calculated by expanding the detected sites to a 20-bp upstream and downstream window.

### 5.6 The correlation analysis of m^6^A levels detected in NP-m^6^A-Finder and GLORI v2.0

The accuracy of methylation level measurement was estimated by computing the Pearson correlation: (i) between two NP-m^6^A-Finder replicates, and (ii) between NP-m^6^A-Finder and GLORI v2.0.

The correlation of NP-m^6^A-Finder m^6^A levels between two HEK293T replicates (Chan et al., 2024) was calculated with the reproduced sites with read coverage ≥20. Correlation analysis was performed using an in-house R script.

For cross-method comparison of m^6^A levels in NP-m^6^A-Finder with GLORI v2.0, an additional reliability filter was applied to NP-m^6^A-Finder based on the average number of m^6^A-positive 5-mer intervals spanning a given site per read. Because our sliding-window approach interrogated each adenosine within five overlapping 5-mers, a truly modified site was expected to be supported by multiple positive intervals. We therefore set up the threshold that the mean number of m^6^A-containing 5-mers per read at a site is > 2, reflecting high-confidence modification calls. The correlation of m^6^A levels in these filtered NP-m^6^A-Finder sites was then computed with GLORI v2.0 measurement restricted to the sites with the coefficient of variation (CV) < 0.05 across its three biological replicates. Furthermore, to look at sequence context-dependent m^6^A quantification correlations, we computed Pearson correlations between NP-m^6^A-Finder and GLORI v2.0 in subsets of the sites residing in distinct DRACH motif contexts (e.g., GGACU, AGACT, etc.), revealing how quantification accuracy varies across varying sequences.

### 5.7 Prediction of m^6^A modifications at the poly(A) tail

We used the aligned SAM files to identify the ranges of poly(A) regions in *T. brucei* (ERR7889820 and ERR7889821) and human HEK293 cells. For each read, the part that failed to align to the reference genome was identified as poly(A). We marked down the start and end positions of poly(A). We also identified the exact gene of the poly(A) of each read by aligning the reads to reference cDNAs. Then, using a slide window of 5 bp with step length 1, we extracted all 5-mers in the poly(A) tail. Features of these 5-mers were extracted, encoded, and segmented as described above. With these features, we identified whether the 5-mers of poly(A) contained m^6^A or not using hard-voting to integrate the prediction results of the trained XGBoost model and RF model. The threshold possibility of positive predictions by the trained models was set to 0.7 instead of 0.5, so that the false positive rate could be decreased. To present the extent of m^6^A modifications of each poly(A) tail, we used the total number of 5-mers containing m^6^A to divide the total number of 5-mers extracted from the poly(A), which was described as the poly(A) level of each read. We also compared the average poly(A) levels between *Trypanosoma brucei*’s m^6^A-containing transcripts (VSG2 reads) and m^6^A-not-containing transcripts (Schwartz et al., 2013), as well as between HEK293 WT cell transcripts and shMETTL3 cell transcripts. To evaluate the performance of our hard-voting ensembled XGBoost and RF model in classifying 5-mers with A polymers, which are dominantly presented in the poly(A) region, we selected GLORI v2.0 annotated m^6^A sites, which are within a sequence context of “AAAAA” and of site m^6^A level averaged from three replicates higher than 70% using in-house Python scripts. Then, we extracted the 5-mers covering these sites from the BAM files generated by minimap2 hg38 genome alignments of the HEK293T DRS data (WT1, WT3, Mettl3 KO1, and Mettl3 KO2) we used before (Chan et al., 2024). The WT-derived 5-mers were labeled as “1” (with m^6^A), while the Mettl3-KO-derived 5-mers were labeled as “−1” (with no m^6^A). These labeled 5-mers were then sent for model evaluation, calculating the accuracy, precision, recall, and F1-score of the ensembled model. The ROC curve of the prediction result was also plotted using in-house Python scripts.

## Data Availability

The original contributions presented in the study are publicly available. All original trained models were included in Google Drive: https://drive.google.com/drive/folders/1w7Ff1Ay76DVXK-Mk_Mi9LF1csrQqcGgq?usp=drive_link. All in-house codes were submitted to GitHub: https://github.com/Cynthia0411/Nanopore_m6A_Finder. Further inquiries can be directed to the corresponding author.
